# Obesity as a Risk and Severity Factor in Rheumatic Diseases (Autoimmune Chronic Inflammatory Diseases)

**DOI:** 10.3389/fimmu.2014.00576

**Published:** 2014-11-11

**Authors:** Elisa Gremese, Barbara Tolusso, Maria Rita Gigante, Gianfranco Ferraccioli

**Affiliations:** ^1^Division of Rheumatology, Institute of Rheumatology and Affine Sciences, Catholic University of the Sacred Heart, Rome, Italy

**Keywords:** rheumatic diseases, body mass index, obesity, adipokines, inflammation, rheumatoid arthritis

## Abstract

The growing body of evidence recognizing the adipose tissue (AT) as an active endocrine organ secreting bioactive mediators involved in metabolic and inflammatory disorders, together with the global epidemic of overweight and obesity, rise obesity as a hot topic of current research. The chronic state of low-grade inflammation present in the obese condition and the multiple pleiotropic effects of adipokines on the immune system has been implicated in the pathogenesis of several inflammatory conditions including rheumatic autoimmune and inflammatory diseases. We will discuss the main relevant evidences on the role of the AT on immune and inflammatory networks and the more recent evidences regarding the effects of obesity on the incidence and outcomes of the major autoimmune chronic inflammatory diseases.

## Introduction

In the last few years, the interest and research in the field of obesity have esponentially risen, due to its increased prevalence and to the burden of the obesity-related diseases (1).

Moreover, white adipose tissue (WAT) has emerged as an active endocrine organ, playing a role not only on metabolism but also on immune and inflammatory processes by releasing a plethora of adipocytokines and pro-inflammatory mediators, among which TNF-α, IL-6, adiponectin, leptin, resistin, visfatin, and C-reactive protein (2).

The inflammatory role of the adipose tissue (AT), together with the rise in incidence of autoimmune diseases, has growing a huge interest in the relationship between obesity and chronic inflammatory diseases.

Obesity in turn predisposes to metabolic and cardiovascular diseases, and it is becoming clear that the dietary habits in Western societies (“too much,” “too fatty,” “too salty”) and a high body mass index (BMI) constitute risk factors for autoimmune diseases (3).

## Definition and Prevalence of Obesity in Rheumatic Diseases

Overweight and obesity are defined by the World Health Organization (WHO) as abnormal or excessive fat accumulation that presents a risk to health (4). BMI was developed as a clinically measurable approximation for body fat percentage and according to the National Institute of Health (NIH) classification, BMI was categorized into three classes, as a BMI <25 Kg/m^2^, identifying normal-weight status, BMI 25–30 Kg/m^2^, identifying overweight and BMI >30 Kg/m^2^, identifying obese status (5).

Body mass index is recognized as a valid measure of absolute fat mass adjusted for height, yet the use of BMI may present some limits. First, the abdominal fat distribution has been suggested being more stringently associated with cardiovascular risk and with AT effects, so anthropometric measures such as waist circumference has been shown to better reflect central or abdominal adiposity than BMI. Waist circumference is accepted as a clinical measure of abdominal obesity and has been proposed as substantial indicator of health risk and as a criteria for the metabolic syndrome (6). Moreover, the accuracy of BMI in estimating the amount of fat mass may vary according to age, as in the older age the lean body mass tends to decrease in favor to adipose mass, to gender, as female had higher proportion of AT, and under some inflammatory disease condition that may alter the body mass composition, like rheumatoid arthritis (RA).

Indeed, methods allowing for the characterization of body fat proportion (e.g., dual energy x-RAY absorpsiometry-DXA, bioelectrical impedence) demonstrated that RA patients have more body fat for a given BMI than healthy controls; moreover, male RA patients had more visceral fat and female more subcutaneous fat compared with controls with similar BMI and waist circumference, so lower BMI cut-offs has been proposed to improve the predictive value of this indicator in RA (7, 8).

However, at present BMI still remains a valid and easily evaluable surrogate to estimate total body fat mass in clinical evaluation (9).

**Figure 1:**
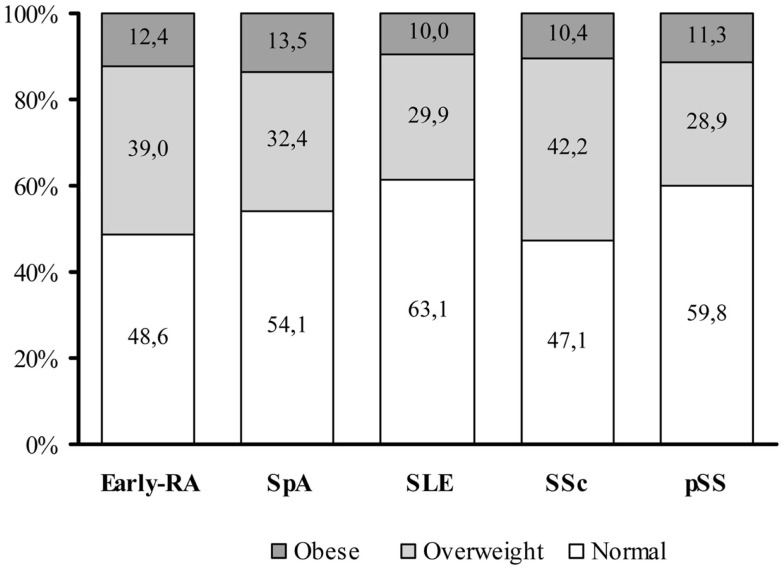
**Distribution of BMI categories among an Italian cohort of adult autoimmune disease patients**. In our tertiary referral centre, the prevalence of overweight [defined according to the WHO International standards as a body mass index (BMI) of 25–29.9] or obesity (BMI ≥30) is similar between the different autoimmune disease categories. The graph summarized data from an early-rheumatoid arthritis (early-RA) cohort of patients (*N* = 401, 76.3% female, mean age: 54.6 ± 14.0 years, mean BMI of 25.3 ± 4.4 Kg/m^2^), a Spondyloarthritis (SpA) cohort (*N* = 220, 30.6% female, mean age: 39.5 ± 11.8 years, mean BMI of 25.1 ± 4.4 Kg/m^2^), a Systemic Lupus Erythematosus (SLE) cohort (506 patients, 90.3% female, mean age: 44.1 ± 14.8 years, mean BMI of 24.0 ± 4.6 Kg/m^2^), a Systemic Sclerosis (SSc) cohort (442 patients, 88.2% female, mean age: 58.5 ± 14.2 years, mean BMI of 24.4 ± 4.8 Kg/m^2^), and a primary Sjogren Syndrome (pSS) cohort (204 patients, 96.1% female, mean age: 56.4 ± 14.3 years, mean BMI of 24.3 ± 4.3 Kg/m^2^) (10). Data are reported as percentage (%) of normal weight, overweight, and obesity in the different diseases.

To date, the prevalence of obesity and overweight in autoimmune chronic inflammatory diseases (ACIDs) are not exhaustively reported and may widely varies depending on the populations considered, the type of disease and the disease duration at the time of evaluation, because the disease and therapy can modify the body weight and composition. Overall, the prevalence of obesity in many rheumatic diseases seems to be similar to slightly higher than the general population, with the exception of Psoriatic Arthritis (PsA). Figure 1 illustrates the distribution of normal weight, overweight, and obese subjects in different cohorts of patients with the most representative inflammatory, autoimmune, and vascular rheumatic diseases in our tertiary referral center. In particular, the prevalence of obesity was 12.4% in patients with early Rheumatoid Arthritis (ERA), 13.5% in seronegative spondyloarthritis (SpA), 10% in Systemic Lupus Erythematosus (SLE), 10.4% in Systemic Sclerosis (SSc), and 11.3% in primary Sjogren syndrome (pSS).

## Obesity and Inflammation

Adipose tissue in normal-weight subjects is composed mostly of adipocytes, i.e., the mature fat cells, and of the interadipocytes stromal vascular fraction including preadipocytes, i.e., the immature adipocyte precursors, fibroblasts, endothelial, and immune cells. Almost the full spectrum of immune cell types, and in particular macrophages, are represented among these AT-resident immune cells, and they play important housekeeping functions ranging from apoptotic cell clearance to extracellular matrix remodeling, tissue homeostasis maintenance, and angiogenesis (11).

The progressive and excessive fat accumulation that occurs in obesity leads to substantial changes in the amount and phenotype of the AT-resident immune cells, with an increasing in number and activity of some of them (most notably macrophages, mast cells, neutrophils, and T and B lymphocytes) while reducing others, including eosinophils and several subsets of T lymphocytes [T helper 2 (Th2), Treg, and iNKT cells] (12). This imbalance contributes to the development of obesity-related local and systemic inflammation and although most types of immune cells are already present in the AT, their number increases notably with the progression of obesity.

In particular, in obese status, macrophages are the most abundant cell type infiltrating fat tissue and they polarize from an anti-inflammatory M2 macrophages pattern toward an inflammatory (M1) phenotype characterized by secretion of pro-inflammatory cytokines and expression of nitric oxide synthase sustaining the process of atherogenesis (13).

The link between metabolism and immunity was further corroborated at the intracellular level, with the main inflammatory signaling pathway comprising nuclear factor-kB (NF-kB) and inhibitor of kB kinase-b (IKKB) stimulated in obesity (14). Conversely, genetic deletion of IKKB or the inhibition of this pathway by salicylates was shown to attenuate insulin resistance in both mice and human beings (15). Furthermore, obesity-related inflammation tends to activate also other pro-inflammatory factors including the group of c-Jun N-terminal protein kinases (JNK), while the ablation of JNK protects experimental animals from diet-induced obesity and inflammation (16).

Nowadays, there is a growing body of evidence that AT is a dynamic endocrine organ that releases several bioactive substances, secreted by adipocytes and adipose-resident macrophages, including some pro-inflammatory cytokines, in common with inflammatory diseases such as RA, like TNF-α and IL-6, and specific cytokines, termed “adipokines,” most of which with pro-inflammatory properties accounting for a chronic low-grade systemic inflammation. Of note, these chronic inflammatory signals can have a profound impact on CD4^+^ T cell populations, and it has been shown, in murine studies, that diet-induced obesity can impact specific fat-resident regulatory T cells (Treg) and particularly promote a Th17-biased immunity, partly dependent on IL-6.

The final picture appears to be characterized by an imbalance between Th1 and Th2 stimuli in fat, perhaps through the depletion of Th2 and AT Treg cells, the increase in CD8^+^ and Th1 cells or both. In addition, adipocytes were found to potently activate T-cells in an antigen- and contact-dependent manner that was enhanced in obesity, and MHCII-deficiency attenuated inflammatory and metabolic responses (17), and many studies have confirmed that the polarization state of an AT macrophages (ATM) correlates well with insulin resistance (18).

Of note, in a mouse model of collagen-induced arthritis with a diet-induced obesity, obese mice had an increased risk of developing arthritis and a more severe arthritis than lean mice. Although obesity does not display a pathogenic role in initiating arthritis, it could play an important role in amplifying the inflammation of arthritis through the Th1/Th17 response (19).

## Adipokines and Inflammation

Since the identification of leptin as the first bioactive molecule produced by AT, a growing body of data has accumulated about the plethora of adipokines produced by WAT (pre-adipocytes and mature adipocytes, as well as infiltrated macrophages), as the major contributors to the low-grade inflammatory state associated with obese condition.

The adipokine family has pleiotropic functions, mediating both endocrine and immune effects on multiple organs, most of which promoting metabolic dysfunctions and inflammatory responses. The association between obesity and chronic inflammation has introduced the idea that adipokines may contribute to the pathogenesis of several inflammatory conditions including rheumatic diseases, contributing to the complex network of inflammatory cascade (20, 21).

The role of adipokines in the mechanisms of rheumatic autoimmune diseases is supported by evidences that they exert potent modulatory actions on target tissues involved in rheumatic diseases including synovial, cartilage, bone, and immune cells. On the other hand, even if adipokines are produced predominantly by AT, they might also be expressed in the joints by chondrocytes, synoviocytes, and immune resident cells (22).

The characteristics and effects of more common adipokines are yet extensively described (23). Here, we briefly summarize the stronger evidences in understanding the role of the most relevant and most widely studied adipokines, i.e., adiponectin, leptin, resistin, visfatin, in the complex network of chronic inflammatory states and autoimmune rheumatic diseases.

### Adiponectin

Adiponectin is almost exclusively secreted by adipocytes and show anti-inflammatory, insulin-sensitizing, and anti-atherogenic properties (24). Obese people displayed low adiponectin plasma levels, suggesting that functional adipocytes in lean subjects produce high adiponectin levels, while in the dysfunctional adipocytes of obese individuals its expression is down-regulated.

On the side of inflammation, several “anti-inflammatory effects” of adiponectin have been reported. It modulates the activity of immune innate response by inducing the production of relevant anti-inflammatory factors such as IL-1 receptor antagonist and IL-10, suppresses TNFα production, promotes the anti-inflammatory M2-phenotype macrophages polarization; also on adaptive immunity, adiponectin acts as a potent modulator of both B and T cells (25).

While in metabolic diseases, the role of adiponectin is clearly anti-inflammatory in rheumatic inflammatory diseases and its role is controversial. Unexpectedly, accumulating evidences on RA found that adiponectin was elevated in RA serum and synovial fluid, it is strongly expressed also at the sinovium level, directly correlate with disease activity and radiologic progression and it seems not affected by TNF blockade (26).

### Leptin

The adipokine leptin, the product of the *ob* gene, is mainly synthesized by WAT and is considered a major regulator of body weight by suppressing appetite, thus decreasing food intake, and stimulating energy expenditure. However, leptin plasma levels are directly correlated with the amount of body fat and obese people have high circulating leptin levels, indicating a resistance to its action in obese status (27).

Leptin is considered a pro-inflammatory cytokine, as its synthesis is enhanced during acute infection and inflammation, as well as under the action of inflammatory mediators such as TNFα, IL-6, and IL-1 (28). Furthermore, leptin itself stimulates the production of pro-inflammatory cytokines from macrophages and increases the production of Th1 type cytokines, thus polarizing the Th1/Th2 balance toward a Th1 phenotype (29, 30).

Despite its pro-inflammatory profile, data on the role of leptin in RA remain controversial. In fact, there are studies with conflicting results regarding differences in circulating leptin levels between RA patients and controls and the association of leptin levels with disease activity (31), even if leptin seems to have a protective role against bone damage (32), as well as in animal models it is not clearly defined whether leptin has inducing or attenuating effects on arthritis development and severity (33).

### Resistin

Resistin was initially thought related to insulin resistance studies in animal models; although evidence for this effect in human beings is less clear (34). Even if resistin production was originally described to be restricted only to WAT, subsequently it was observed that in human beings resistin mainly derives from circulating monocytes and macrophages (35), suggesting a major role in inflammatory processes in human beings.

In this regard, several studies demonstrated that resistin expression is up-regulated by pro-inflammatory cytokine, and in turn, resistin is able to stimulate the production of TNFα, IL-6, IL12 TNF-α, IL-1β, and resistin itself (36). These evidences, together with the finding that the injection of resistin into mice joints triggers synovitis, highlight a potential role of resistin in the inflammatory cascade of inflammatory arthritis (36). In fact, in RA, serum, and synovial fluid resistin levels were higher than in osteoarthritis, as well as resistin expression in synovial lining layers. Moreover, resistin levels positively correlated with acute phase reactants and RA disease activity and seem modulated by anti-TNF therapy (37).

### Visfatin

Visfatin, initially named pre-B-cell colony-enhancing factor, is an adipokine with insulin-mimetic actions, preferentially produced by visceral AT (38). Circulating levels of visfatin correlates with the amount of visceral fat and are increased in patients with obesity and type-2 diabetes and are reduced after weight loss (39).

Like resistin, visfatin is synthesized also in response to inflammatory stimuli and, on the other hand, it can enhances production of TNF-α, IL-6, IL-1β, as well as of IL-10 and IL-1Ra (40). Additionally, it promotes activation of T cells by enhancing the expression of co-stimulatory molecules, such as CD40, CD54, and CD80, on monocytes, acts as a chemotactic factor on monocytes and lymphocytes and it strongly affects the development of both T- and B-lymphocytes. As in other inflammatory conditions, in RA circulating levels of visfatin are increased, as well as its expression in synoviocytes at sites of attachment and invasion into cartilage or bone. In agreement with these findings, it is the association between radiographic damage and high visfatin serum concentrations. Visfatin serum and synovial fluid levels correlated with the degree of inflammation, with the severity of the disease, and with joint damage. Moreover, inhibition of visfatin in collagen-induced arthritis mice reduced arthritis severity with similar effect to that produced by TNF-α inhibitor (41).

## PEDF and Chemerin as Inflammatory Adipokines in Obese Patients with Autoimmune Diseases

The adipokine family is continuously growing and among the emerging adipokines, pigment epithelium-derived factor, and chemerin seem to be key players in linking obesity and inflammation in rheumatic diseases.

Pigment epithelium-derived factor (PEDF or SERPINF1) has been described as one of the new adipokines involved in the development of obesity-related disease. PEDF is a 50 kDa secreted glycoprotein, member of the serpin (serine protease inhibitor) family lacking the serine-reactive loop and thus has no function on protease inhibition (42, 43). The PEDF protein was originally identified in retinal cell culture supernatants and characterized as a neurotropic factor (44), but recently, it has been associated with anti-tumor effects based on its anti-angiogenesis and pro-apoptosis activities (45). On the other hand, PEDF leads to insulin resistance and inflammatory signaling in several cell types (46–48).

The SERPINF1 gene is expressed in AT, liver, and bone marrow in human beings. Although the circulating levels of PEDF are thought to derive from the liver (49), to date it is well demonstrated that PEDF is up-regulated during adipogenesis and is mostly produced by mature adipocytes, suggesting that AT contributes to plasma PEDF levels (50, 51). This notion is consistent with studies indicating that PEDF is one of the most abundant protein secreted by human adipocytes or from human mesenchymal stem cells (48, 52). This direct association between serum PEDF levels and central obesity or the other components of the metabolic syndrome was well documented (47, 53–55).

Furthermore, in genetically and diet-induced obese mice, AT PEDF expression and plasma PEDF levels increased up to threefold whereas liver and skeletal muscle expressed only low amounts of PEDF, which did not increase upon obesity. In caloric restriction diet-induced obese mice, a reduction in AT PEDF expression was demonstrated (46).

In addition to its role in neurogenesis and angiogenesis, PEDF induces inflammatory signals in several cell lines and circulating PEDF levels correlate with the inflammatory burden and vascular dysfunction in individuals with type 1 diabetes (45, 52).

Consistent with this notion, the pathophysiology of obesity is characterized not only by the increased expression of inflammatory cytokines such as interleukin (IL)-6 and tumor necrosis factor (TNF)-α but also by the higher expression of PEDF and long-term obesity may inhibit regulatory responses resulting in a systemic pro-inflammatory state. Little is known about the regulation of PEDF expression and secretion but there is evidence of a possible involvement of hypoxia in human adipocytes (56).

Moreover, Chavas et al. demonstrated that PEDF directly activates inflammatory signaling proteins, p38 MAPK and ERK1/2, in macrophages, and activation of these inflammatory kinases was required for PEDF-mediated macrophage activation (57). In addition, PEDF mediates activation of pro-inflammatory signaling proteins, p38 MAPK and NF-κB, in muscle and fat cells in culture. Although PEDF has been considered as a major factor in the adipocyte culture contributing to macrophage activation, it is plausible that other factors, such as IL-1 or adipokines, may also lead to macrophage activation and synergistically enhance TNF release (48). On the contrary, the protein deacetylase sirtuin-1 (SirT1) plays a role in the repression of inflammation in AT, via suppression of JNK and NF-kB signaling disrupting adipocyte-macrophage communication (58, 59).

The inflammatory milieu of obesity is complex, featuring a panoply of elevated plasma and tissue specific cytokines that are increased in some rheumatic diseases and are able to increase the expression of inflammatory cytokines, such as TNF and IL-6, also in the early phases of the disease. Moreover, obesity is associated in some studies with an increased risk of rheumatic diseases (60, 61); and recent reports have showed a negative association between high BMI and response to anti-TNF agents in both RA and SpA (62–64), suggesting that fat mass may affect the response to biologic agents. Pathophysiological mechanisms by which BMI influences anti-TNF biological drugs response remain still unclear.

To support the possible role of PEDF as a player in inflammatory burden of RA, we observed that the circulating PEDF levels, evaluated in a cohort of patients with early RA at the time of RA diagnosis, are higher in obese and overweight than in normal-weight subjects and correlated with systemic inflammation (ESR and CRP) (65).

In recent years, another adipokine, named chemerin, has emerged has a key adipokine involved in inflammation and immune responses in RA. Chemerin is involved in adipogenesis, chemotaxis, and activation of dendritic cells and macrophages. In healthy conditions, the overweight and obese subjects have higher levels of circulating chemerin compared to normal-weight subjects, which decrease after reduction of fat mass as a result of a dietary route (66, 67).

Chemerin is found in inflamed tissues and biological fluids of different inflammatory diseases, such as RA. Recent studies have shown that synovial fibroblasts (FLSs) of RA patients express chemerin and its receptor (ChemR23) and that circulating levels of this molecule directly correlate with disease activity (68, 69). The expression of ChemR23 on RA fibroblasts and chemerin itself activate FLSs to enhance the production of IL-6 and CCL2 (70–72). Therefore, these observations indicate that chemerin may be involved in the enhancement of local pro-inflammatory cytokine and chemokine production by RA FLSs, leading to persistent amplification of inflammation in the RA synovium, possibly in an autocrine or paracrine manner. Recently, Ha et al. found that chemerin levels directly correlate with disease activity in RA (73). However, it is not yet completely clear whether the circulating levels of chemerin in patients with RA are more associated with systemic inflammation or AT itself, but it is confirmed its role as a biomarker of disease activity.

In this regard, adipokines, i.e., PEDF and chemerin, may provide a metabolic link between obesity and RA or other autoimmune diseases, and as such they could be possible biomarkers of the effect of weight loss and of the decreased fat tissue in chronic inflammatory diseases. In fact, new evidences has emerged to suggest that weight loss may lead to an improvement of quality of life and a reduction of inflammation and of systemic inflammatory mediators in patients with obesity and RA after weight loss and metabolic control with a dietary intervention.

## Impact of Obesity on Autoimmune Chronic Inflammatory Diseases

### RA and obesity

Obesity in RA was found prevalent in 18–31% of patients, overall slightly higher than in the general population. An overweight condition has been noted in more than 60% of RA patients (74, 75).

#### RA incidence

In the past years, several studies evaluated the impact of obesity on RA incidence, but with inconclusive results, probably due to small size number of patients included and to the low prevalence of obesity (61, 76–78). More recently, a large retrospective case–control study showed that obesity is associated with a modest risk of developing RA (OR:1.24; 95% CI: 1.01, 1.53 adjusted for smoking status), but about 50% of the increasing incidence of RA among this population from Minnesota could be attributed to the obesity and this effect is more pronounced in woman (79).

Moreover, data from three different cohorts of patients seem to indicate that obesity is associated with a likelihood of developing a seronegative RA, with an OR between 1.6 and 3.45 (78, 80, 81). In particular, in a large Swedish case–control study, dividing patients according to the ACPA (anti-citrullinated protein antibody) positivity, the RA risk linked to obesity appears different between genders; in fact, only the female gender was associated with the occurrence of ACPA-negative RA and there was no association between obesity and ACPA-positive RA among women, while an inverse association between BMI and ACPA-positive RA was seen in men (80).

In a recent study involving two large prospective cohorts of women (Nurses’ Health Study-NHS 109,896 woman and Nurses’ Health Study II-NHSII, 108,727 woman, with 1,181 incident cases of RA), Lu et al. observed a significant association between being overweight and obese and developing RA, both in seropositive and seronegative subset. This association was even stronger among women diagnosed at younger ages, i.e., ≤55 years, with a 35% increased risk of developing RA, and an almost 50% increased risk of developing seropositive RA in adulthood. Interestingly, also the “exposure time” to obesity seems to influence the RA risk in woman aged 55 or younger with a 37% increased risk of RA with a history of 10 years of being obese (82).

#### RA activity and severity

Considering the effects of obesity on RA activity and severity, published data suggest a negative influence of a high BMI on disease activity. In a large cohort of early RA followed-up for an average of 9.5 years, at the study inclusion obese subjects had slightly higher HAQ, VAS pain and global health scores than patients with a BMI <30, with similar DAS28 and systemic inflammatory indexes (ESR, CRP). In the same cohort, at the last follow-up, obese people had a more active and severe disease, with a decreased probability of being in disease remission and of reaching sustained remission, despite similar use of DMARDs, steroids, and biologic drugs in different BMI groups (83). The association between worst disease activity and disability was confirmed also in other cohorts of patients with established RA (84, 85).

In a sub-analysis of the BeST study evaluating the effects of a BMI >25 on response to therapy in patients with early RA (disease duration <3 years) naïve to DMARDs, subjects with a BMI under or over 25 had no differences in baseline DAS, inflammatory parameters, HAQ, pain, and global health assessment (86). In our cohort of 346 early RA with symptoms duration <12 months, we observed that disease activity indexes (DAS and DAS28) were higher in obese and overweight patients compared to normal-weight patients (87).

Regarding disability, like in the general population, worsening functional capacity and quality of life has been linked to increased fat mass and, both in the general population and in patients with RA, pain prevalence, and severity have been linked to obesity; pain in RA has a large impact on self-reported health, physical function, and disease activity index (88, 89).

Moreover, it can be hypothesized that adiposity may influence also the evaluation of swollen joint, but Caplan et al. showed that the swollen joint count performs in obese subjects at least as well as in subjects with lower BMI (90). Moreover, as previously discussed, the worst disease activity and severity in obese RA patients can be derived from the close association of obesity with activation of pro-inflammatory pathways.

Despite the evidences of a more severe disease, data on radiographic progression suggest that obesity may be protective against joint damage (91–94). Studies in patients with early RA, of up to 3-year duration, suggest that the protective effect of high BMI seems to be present before the diagnosis of RA, with overweight and obese RA patients exhibiting less joint damage than their normal-weight counterparts at the time of diagnosis; this effect appears to continue during the first years of RA, with joint damage progressing less rapidly in obese than in normal-weight RA patients (94). The possible reasons for this finding may include an increase mechanical load stimulating bone synthesis, as suggested for the protective effect of obesity from osteoporosis development in the general population, a lower joint destruction in seronegative RA, the RA subset more associated with obesity, higher levels of estrogens found in obese subjects, having a bone protective effects, and role of adipokines, in particular adiponectin. As previously discussed, adiponectin might induce disease activity in the joint, resulting in more active disease in lean having higher adiponectin levels and less active in obese patients that displayed lower adiponectin levels (95). However, this finding has to be further clarified.

#### Obesity and RA response to therapy

Regarding the impact of high BMI on response to RA treatments, all the published studies showed that obesity represents an additional risk of poor response to therapy and of not reaching remission. In first studies conducted on long standing RA, obese patients were 50% less likely to obtain disease remission after 12 months of a first-line anti-TNF treatment than normal-weight subjects, and the worst outcome was observed with the weight-dosed drug, i.e., infliximab (62, 63, 96).

Similar results were confirmed by observational studies including early-RA patients, in whom both obesity and overweight confer a lower chance to obtain remission or low disease activity, irrespective of treatment with DMARDs or anti-TNF therapy (86, 97, 98).

Moreover, more than twice overweight and obese subjects with Early-RA needed anti-TNF therapy after 12 months from diagnosis, compared to normal-weight patients (87).

The reason why obesity affects the RA outcomes, in particular in patients treated with infliximab needs to be clarified. Mechanisms that can be postulated are a direct influence of obesity on drug pharmacokinetics and an inflammatory and therapy-resistant state induced by AT by the release of specific adipocytokines that are increased in RA patients and are able to increase the expression of inflammatory cytokines, such as TNF and IL-6. In this regard, however, studies testing the effect of TNF blockade on adipokine plasma levels in patients with RA are not conclusive, and the majority of the studies show that anti-TNF drugs have no influence on the levels of adipocytokines (99). However, the AT may be associated with an induction of resistance to all of the anti-TNF drugs, and understanding its role requires further research. Regarding infliximab, it has recently been shown that Fc receptors modulate the efficacy of infliximab both *in vitro* and *ex vivo*, whereas the presence of this receptor has no impact on the inhibitory activity of certolizumab-pegol, which lacks the Fc fragment (100). These data could be helpful in the light of the fact that omental adipocytes show high expression of Fc receptors (101).

### SpA and PsA and obesity

Seronegative SpA are a heterogeneous group of inflammatory diseases including ankylosing spondylitis (AS), PsA, inflammatory bowel disease associated SpA (EnteroSpA), and undifferentiated SpA (USpA), sharing some clinical characteristics, in particular the involvement of axial spine. At present, only a very few studies has evaluated the impact of body weight on SpA, focusing on axial involvement. In a cohort of 155 patients affected by AS starting treatment with Infliximab at the dosage of 5 mg/Kg, Ottaviani et al. reported that the response to therapy (BASDAI50) at 6 months was significantly lower in obese (26.5%) than in normal-weight (77.6%) individuals (64). In another study involving 170 patients affected by a form of SpA with axial involvement treated with a first-line anti-TNF therapy, the proportion of axial SpA patients reaching the BASDAI50 after 12 months fell from 72.8% in normal weight to 54.5% in overweight and 30.4% in obese subjects (102); the lower response rate according to BMI was obtained under Infliximab treatment.

Psoriatic arthritis, although part of the SpA group, deserves a separate discussion. More extensive studies are available on PsA and psoriasis, conditions associated with a high prevalence of metabolic syndrome and other metabolic disorders. Both PsA and psoriasis has been associated with an enhanced prevalence of obesity and overweight. A recent meta-analysis including 201,831 psoriatic patients on a total of 2.1 million subjects conclude that psoriatic patients are at significantly higher risk of obesity compared with the general population, with an OR from 1.46 for patients with mild psoriasis to 2.23 for severe-psoriasis (103).

Bhole et al. evaluating patients with PsA, psoriasis, and RA, found that in all these conditions mean BMI and prevalence of obesity were higher compared to the general population, in which BMI was 26.1 and obese people 18%. Interestingly, BMI values were similar between RA and psoriasis patients, while PsA patients have higher BMI values than those with psoriasis or RA (29.6, 27.9, and 27.3 Kg/m^2^, respectively) and the prevalence of obesity was 37% in PsA, 29% in psoriasis, and 27% in RA (104).

The effects of these two metabolic and autoimmune disorders are probably bidirectional, as there are evidences supporting that obesity may be a risk factor predisposing to the development of psoriasis and PsA, while others suggesting that overweight could be a consequence of these conditions rather than a predisposing factor. However, obesity is a well recognized risk factor for the development of psoriasis and PsA. In a large population of 75,395 individuals with psoriasis, obesity has been associated with a high risk of incident PsA (105).

Only few studies assessed the relationship between obesity and the severity of arthritis in patients with PsA. Di Minno et al. reported that increased BMI predicted less favorable response to TNFα blockers in patients with PsA who were followed for 24 months (106); and Eder et al. found that overweight and obesity are associated with a lower probability of achieving MDA among patients with PsA, independently of the use of biological and non-biological DMARDs (107).

Of particular interest and of crucial clinical relevance is the effect of weight loss on inflammation and disease outcome. The few studies evaluating interventions aiming at lowering the body weight showed significant improvement of psoriasis or PsA severity, as well as the cardiovascular risk profile in both diseases. Weight reduction was also associated with improved response to treatment with TNFα blockers (108–110).

## Connective Systemic Tissue Diseases: SLE, SSc and pSS and Obesity

The prevalence of obesity in SLE is between 28 and 50% (111, 112). Some studies investigated the impact of BMI on SLE, evidencing conflicting results about a relationship between BMI and the disease manifestations (113–115). Data from the different cohorts showed that about two-thirds of patients with SLE were either overweight or obese but contrasting data have been reported about an association between overweight and obesity and disease activity in SLE patients (116). On the other hand, an increased BMI has shown to be associated with older age, less social support, depression, poorer self-reported QoL, and fatigue in patients with SLE (117).

It has been well established that SLE is associated with an increased risk of cardiovascular disease as compared with general population and the Framingham cardiovascular risk score was significantly higher in SLE women classified as obese compared to normal-weight patients. Increased waist circumference increases the risk of atherosclerosis and the incidence of arterial and venous thromboembolism in patients with SLE (118, 119).

Finally, there was a statistically significant association between BMI in the SLE patients and the presence of lupus nephritis and hypertension (120). This is important because hypertension is a strong risk factor for cardiovascular disease, which is one of the major causes of death in SLE (121, 122).

The prevalence of obesity in SSc is between 9 and 18%. This lower percentage of obesity in SSc than in normal population was observed mainly in women with diffuse SSc, but not in those with limited SSc (123). In SSc patients, visceral abdominal fat has been correlated with cardiovascular risk factors and lung functionality, as already found in a large population of elderly men and women (124–126).

Considering that SSc is a form of micro- and macro-vascular angiopathy the presence of obesity should be regarded as an additive factor in terms of cardiovascular risk. On the contrary, a low BMI, together with age ≥65 years, low forced vital capacity (<50% of the predicted value), clinically significant arrhythmia on electrocardiogram, absence of anticentromere antibodies, hypertension, chest radiograph suggestive of pulmonary fibrosis, has been demonstrated to be a predictor of mortality in early SSc (127).

To date, no data are reported about the involvement of obesity in pSS and its effects. A recent study on a multicentre cohort of UK pSS patients demonstrated that pain and depression were the most important predictors of the health-related quality of life in patients with pSS, accounting for 48% of the variability. Moreover, BMI, fatigue, and anxiety had been identified as significant predictors, but accounting for <5 of the variability in terms of EuroQoL-5 dimension utility values (128).

## Conclusive Remarks

Obesity is a risk factor for many inflammatory and autoimmune diseases, in terms of incidence, disease severity, and outcomes, as well as for the overall cardiovascular risk.

Research in the field of obesity and its related effects is continuously growing and the knowledge of these issues has increased significantly in recent years. Understanding even more deeply the pathophysiologic mechanisms underlying the interaction between obesity, inflammation, and disease pathways appears to be of upmost importance to improve the outcomes in patients with rheumatic diseases.

This overview provides a rationale for further studies on the role of AT in RA and the mechanisms behind weight changes during the course of RA disease, as well as the potential effect of weight control on the efficacy of medication disease severity, and complications.

Evaluating the effects of weight loss on the inflammatory/autoimmune disease course appears to be crucial in terms of potential clinical and also pharmacoeconomics perspectives.

## Conflict of Interest Statement

The authors declare that the research was conducted in the absence of any commercial or financial relationships that could be construed as a potential conflict of interest.
